# Encephalopathy at admission predicts adverse outcomes in patients with SARS‐CoV‐2 infection

**DOI:** 10.1111/cns.13687

**Published:** 2021-06-16

**Authors:** Lei Tang, Shixin Liu, Yanhe Xiao, Thi My Linh Tran, Ji Whae Choi, Jing Wu, Kasey Halsey, Raymond Y. Huang, Jerrold Boxerman, Sohil H Patel, David Kung, Renyu Liu, Michael D. Feldman, Daniel D Danoski, Wei‐hua Liao, Scott E. Kasner, Tao Liu, Bo Xiao, Paul J. Zhang, Michael Reznik, Harrison X. Bai, Li Yang

**Affiliations:** ^1^ Department of Neurology Xiangya Hospital Central South University Changsha China; ^2^ Xiangya School of Medicine Central South University Changsha China; ^3^ Department of Diagnostic Imaging Warren Alpert Medical School of Brown University Providence RI USA; ^4^ Department of Radiology The Second Xiangya Hospital Central South University Changsha China; ^5^ Department of Radiology Brigham and Women's Hospital Boston MA USA; ^6^ Department of Radiology University of Virginia Charlottesville VA USA; ^7^ Department of Neurosurgery Hospital of the University of Pennsylvania Philadelphia PA USA; ^8^ Department of Anaesthesiology and critical care medicine Hospital of the University of Pennsylvania Philadelphia PA USA; ^9^ Department of Pathology and Laboratory Medicine Hospital of the University of Pennsylvania Philadelphia PA USA; ^10^ Department of Radiology Xiangya Hospital Central South University Changsha China; ^11^ Department of Neurology Hospital of the University of Pennsylvania Philadelphia PA USA; ^12^ Department of Biostatistics and Public Health Brown University Providence RI USA; ^13^ Department of Neurology Warren Alpert Medical School of Brown University Providence RI USA; ^14^ Department of Neurology The Second Xiangya Hospital Central South University Changsha China

**Keywords:** encephalopathy, COVID‐19, SARS‐CoV‐2, neurologic symptoms

## Abstract

**Aims:**

To determine if neurologic symptoms at admission can predict adverse outcomes in patients with severe acute respiratory syndrome coronavirus 2 (SARS‐CoV‐2).

**Methods:**

Electronic medical records of 1053 consecutively hospitalized patients with laboratory‐confirmed infection of SARS‐CoV‐2 from one large medical center in the USA were retrospectively analyzed. Univariable and multivariable Cox regression analyses were performed with the calculation of areas under the curve (AUC) and concordance index (C‐index). Patients were stratified into subgroups based on the presence of encephalopathy and its severity using survival statistics. In sensitivity analyses, patients with mild/moderate and severe encephalopathy (defined as coma) were separately considered.

**Results:**

Of 1053 patients (mean age 52.4 years, 48.0% men [*n* = 505]), 35.1% (*n* = 370) had neurologic manifestations at admission, including 10.3% (*n* = 108) with encephalopathy. Encephalopathy was an independent predictor for death (hazard ratio [HR] 2.617, 95% confidence interval [CI] 1.481–4.625) in multivariable Cox regression. The addition of encephalopathy to multivariable models comprising other predictors for adverse outcomes increased AUCs (mortality: 0.84–0.86, ventilation/ intensive care unit [ICU]: 0.76–0.78) and C‐index (mortality: 0.78 to 0.81, ventilation/ICU: 0.85–0.86). In sensitivity analyses, risk stratification survival curves for mortality and ventilation/ICU based on severe encephalopathy (*n* = 15) versus mild/moderate encephalopathy (*n* = 93) versus no encephalopathy (*n* = 945) at admission were discriminative (*p *< 0.001).

**Conclusions:**

Encephalopathy at admission predicts later progression to death in SARS‐CoV‐2 infection, which may have important implications for risk stratification in clinical practice.

## Graphical abstract

Patients with severe acute respiratory syndrome coronavirus 2 infection have a high prevalence of neurologic manifestations, including headache, encephalopathy, dizziness, taste, and smell impairment. Patients with encephalopathy at admission predict later progression to death and mechanical ventilation/ICU admission in SARS‐CoV‐2 infection, which may have important implications for risk stratification in clinical practice.

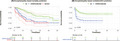

## 1 INTRODUCTION

The outbreak of coronavirus disease 2019 (COVID‐19), caused by novel severe acute respiratory syndrome coronavirus 2 (SARS‐CoV‐2), has been a serious threat to public health. According to the World Health Organization (WHO), there have been 136,996,364 confirmed cases of COVID‐19 and 2,951,832 confirmed deaths in 216 countries, areas, and territories until April 14, 2021. Approximately 20% of patients develop a severe respiratory illness that ultimately requires mechanical ventilation, with mortality rates exceeding 50% in these severe cases.^1^ Due to the high risk of developing critical illness and adverse outcomes and the intensive demands placed on medical resources as the number of severe cases increases, it is critical to identify patients with COVID‐19 who may be more susceptible to advanced disease progression at an early stage, preferably at the time of hospital admission.

Recent studies have shown that severe acute respiratory syndrome coronavirus 2 (SARS‐CoV‐2) infection has a myriad of neurological manifestations.^2^, ^3^, ^4^, ^5^, ^6^, ^7^, ^8^, ^9^ Several studies specifically described a high prevalence of serious neurologic manifestations in patients with severe COVID‐19, which suggests that the nervous system may become more involved as the disease progresses. ^5^, ^9^, ^10^, ^11^, ^12^ Despite the continuously increasing reports of the neurological symptoms of SARS‐CoV‐2, our knowledge about the possible association between early neurologic manifestations and subsequent deterioration leading to intensive care unit (ICU) admission, mechanical ventilation, and death remains unknown. Herein, we will aim to determine associations between encephalopathy and other neurologic manifestations of COVID‐19 and adverse outcomes in a large cohort of hospitalized patients. We will also aim to determine the accuracy of early neurologic manifestations in predicting subsequent adverse outcomes in COVID‐19 patients and then discuss possible routes of SARS‐CoV‐2 nervous system involvement based on the current evidence.

## 2 METHODS

### 2.1 Study design and participants

**FIGURE 1:**
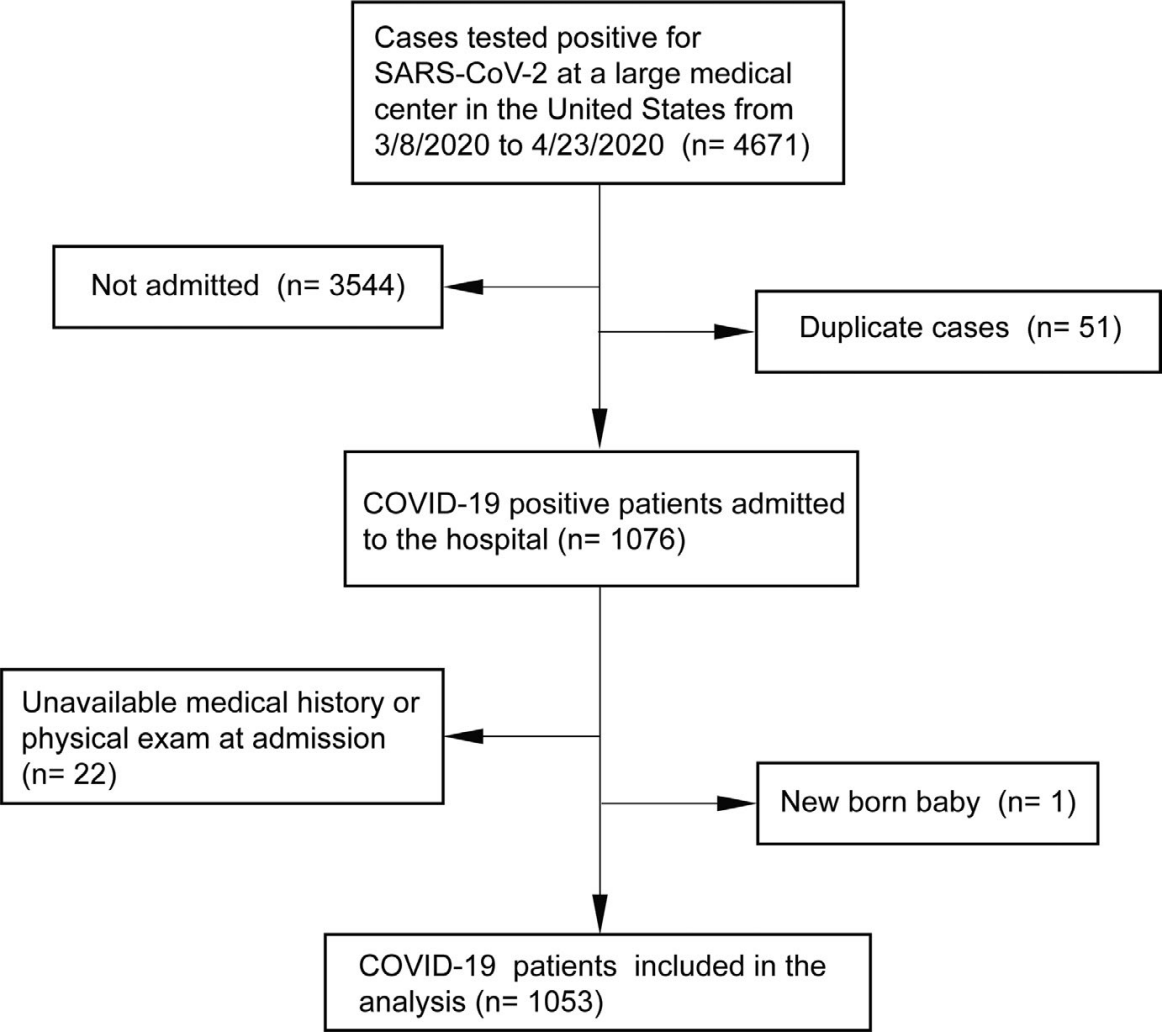
Flow chart of inclusion/exclusion

We conducted a retrospective observational cohort study using data from the hospitals in the University of Pennsylvania Healthcare System (including the Hospital of the University of Pennsylvania, Pennsylvania Hospital, Penn Presbyterian Medical Centre and Chester County Hospital) in the United States. We identified consecutive patients hospitalized with laboratory‐confirmed COVID‐19 infection between March 8, 2020 and April 23, 2020. The diagnosis of COVID‐19 was determined according to WHO interim guidance and confirmed by ribonucleic acid (RNA) detection of SARS‐CoV‐2 in our onsite clinical laboratory. A total of 1053 consecutively hospitalized patients with laboratory confirmation of SARS‐CoV‐2 were included in the analysis (Figure 1).

### 2.2 Standard protocol approvals, registrations, and patient consents

The study was approved and the requirement for informed consent was waived by our medical center's Institutional Review Board.

### 2.3 Data collection

Each patient's electronic medical record was reviewed, with data abstracted from physician and nursing documentation, laboratory results, and radiologic examinations, and recorded in a pre‐specified electronic collection form by two medical students in consensus under the supervision of an attending physician. Collected data included patients’ demographics (age and sex), comorbidities (hypertension, diabetes, cardiac or cerebrovascular disease, malignancy, and chronic kidney disease), typical COVID‐19 symptoms (fever, cough, dyspnea, fatigue, chill, nausea, anorexia, diarrhea, throat pain, chest pain, and abdominal pain), and neurological symptoms. Laboratory results were extracted from medical records on the day of hospital admission (before transferring into ICU). As most patients lack complete laboratory data on the day of admission, only 495 cases were included when analyzing laboratory parameters. Results of all radiologic testing (including brain CT and MRI) were also recorded.

### 2.4 Neurologic symptoms

We distinguished patient symptoms present at the time of hospital admission from subsequent symptoms that occurred later during hospitalization. We specifically focused on encephalopathy as our primary neurologic symptom of interest, but also included other neurologic symptoms related to the central nervous system (CNS) (dizziness/vertigo, headache, stroke, ataxia, aphasia, and seizure) and peripheral nervous system (PNS) (taste impairment, smell impairment, and vision impairment).^5^

Encephalopathy was defined as global disturbance in brain function, which was expressed clinically as either subsyndromal delirium, delirium or coma with possible additional features, such as seizures or extrapyramidal signs.^13^ Encephalopathy was categorized as severe if the patient developed coma, and mild/moderate if the patient did not develop coma.

### 2.5 Outcomes

For each patient, we recorded the timing of hospital admission, discharge, ICU admission, mechanical ventilation, and death, with clinical outcomes followed up to May 26, 2020. We defined mortality as our primary outcome and mechanical ventilation and ICU admission as a combined secondary outcome, and then calculated time to each event in calendar days.

### 2.6 Statistical analysis

Means and standard deviations (SD) were used to describe normally distributed baseline data and median and interquartile ranges to describe non‐normally distributed baseline data. Categorical variables were expressed as counts and percentages. Kolmogorov‐Smirnov test was performed to access the distribution of continuous variables. In this study, all laboratory parameters are subject to non‐normal distribution, and the Mann‐Whitney *U* test was used to assess the statistical difference between the encephalopathy group and the non‐encephalopathy group in laboratory parameters. Chi‐square test was used to compare group differences of categorical variables. Univariable Cox regression analysis based on forward likelihood ratio was performed to evaluate the association of clinical characteristics at admission with later progression to death or mechanical ventilation/ICU admission. Clinical characteristics and laboratory parameters that were significant on univariable analysis were subsequently included in multivariable Cox regression models. Due to missing part of the laboratory data of some patients, we used the following method to fill in the missing data: We first standardized the laboratory data, then filled in the missing values based on the K nearest neighbor algorithm, and finally scaled to the [0,1] interval. We then performed receiver operating characteristic (ROC) analysis with calculation of areas under the curve (AUC) to determine the accuracy of our models in predicting progression to death or mechanical ventilation/ICU admission using variables from our final multivariable Cox regression models. Concordance index (C‐index) for right‐censored data was applied to evaluate the performance of these prediction models.^14^ Finally, we stratified patients into subgroups based on the presence or absence of encephalopathy on admission and compared adverse outcome rates using survival statistics. In a sensitivity analysis, we separately considered patients with mild/moderate and severe encephalopathy.

All statistical analyses were performed using R version 4.0.0 software (the R Foundation). The significance threshold was set at a 2‐sided P value less than 0.05.

## 3 RESULTS

### 3.1 Baseline characteristics and clinical features

We identified a total of 1053 hospitalized patients with confirmed SARS‐CoV‐2 infection over the time period studied. Mean age was 52.4 (standard deviation [SD] 20.2) years, 48.0% (*n* = 505) were male, and 35.1% (*n* = 370) had neurologic symptoms at the time of hospital admission (CNS 31.5% [*n* = 332], PNS 4.8% [*n* = 51]). In patients with CNS symptoms on admission, the most commonly reported were headache (16.3% [*n* = 172]), encephalopathy (10.3% [*n* = 108]), and dizziness (6.3% [*n* = 66]). In patients with PNS symptoms, the most commonly reported were taste impairment (3.8% [*n* = 40]), and smell impairment (3.9% [*n* = 41]).

A total of 174 patients developed encephalopathy during their hospitalization, including 66 that occurred later during hospitalization. Of 108 cases that had encephalopathy on admission, 15 patients were comatose and were characterized as having severe encephalopathy, while 93 patients were non‐comatose. Encephalopathy was predominantly first recorded in documentation from emergency medicine clinicians (Table S1).

Patients with encephalopathy were significantly older and had a higher prevalence of numerous comorbidities compared to patients without encephalopathy, while symptom profiles and laboratory findings also significantly differed between groups (Table 1, Table S2 and Table S3).

**TABLE 1:** Clinical characteristics of COVID‐19 patients with and without encephalopathy at admission

Clinical and laboratory	Total (*n* = 1053)	Encephalopathy (*n* = 108)	No encephalopathy (*n* = 945)	*P* value
Clinical characteristic, no. %				
Age, mean (SD), y	52.4 (20.2)	72.9 (15.3)	50.0 (19.4)	
<50	487 (46.2)	8 (7.4)	479 (50.7)	**<0.001**
≥50	566 (53.8)	100 (92.6)	466 (49.3)	
Sex				
Male	505 (48.0)	48 (44.4)	457 (48.4)	0.440
Female	548 (52.0)	60 (55.6)	488 (51.6)	
Comorbidities				
Hypertension	567 (53.8)	87 (80.6)	480 (50.8)	**<0.001**
Diabetes	349 (33.1)	50 (46.3)	299 (31.6)	**0.002**
Cardiac or cerebrovascular disease	249 (23.6)	59 (54.6)	190 (20.1)	**<0.001**
Chronic kidney disease	164 (15.6)	40 (37.0)	126 (13.3)	**<0.001**
Malignancy	107 (10.2)	21 (19.4)	86 (9.1)	**0.001**
Typical symptoms				
Cough	788 (74.8)	63 (58.3)	725 (76.7)	**<0.001**
Fever	776 (73.7)	91 (84.3)	685 (72.5)	**0.008**
Dyspnea	712 (67.6)	77 (71.3)	635 (67.2)	0.388
Fatigue	448 (42.5)	37 (34.3)	411 (43.5)	0.066
Chill	365 (34.7)	15 (13.9)	350 (37.0)	**<0.001**
Nausea	283 (26.9)	14 (13.0)	269 (28.5)	**0.001**
Diarrheal	271 (25.7)	13 (12.0)	258 (27.3)	**0.001**
Chest pain	250 (23.7)	7 (6.5)	243 (25.7)	**<0.001**
Throat pain	246 (23.4)	8 (7.4)	238 (25.2)	**<0.001**
Anorexia	218 (20.7)	15 (13.9)	203 (21.5)	0.065
Abdominal pain	170 (16.1)	8 (7.4)	162 (17.1)	**0.009**
Nervous system symptoms				
Headache	172 (16.3)	6 (5.6)	166 (17.6)	**0.001**
Dizziness	66 (6.3)	4 (3.7)	62 (6.6)	0.246
Seizure	24 (2.3)	11 (10.2)	13 (1.4)	**<0.001**
Acute cerebrovascular disease	4 (0.4)	1 (0.9)	3 (0.3)	0.352
Ataxia	6 (0.6)	4 (3.7)	2 (0.2)	**0.001**
Taste impairment	40 (3.8)	0 (NA)	40 (4.7)	NA
Smell impairment	41 (3.9)	0 (NA)	41 (4.7)	NA
Vision impairment	2 (0.2)	0 (NA)	2 (0.5)	NA
Outcomes				
Mortality	126 (12.0)	58 (53.7)	49 (5.2)	**<0.001**
Ventilation/ICU	221 (21.0)	66 (61.1)	155 (17.3)	**<0.001**

Abbreviations: SD, Standard deviation. ICU, intensive care unit.

Bold values are statistically significant.

Patients with encephalopathy at admission also had a significantly higher frequency of death, mechanical ventilation, and ICU admission in our univariable analysis, and early encephalopathy was the only significant neurologic symptom associated with mortality (mild/moderate: hazard ratio [HR] 3.005, 95% confidence interval [CI] 2.071–4.360; severe: HR 3.895, 95% CI 1.997–7.594) and ventilation/ICU (mild/moderate: HR 3.333, 95% CI 2.447–4.539) (Table S4). Of note, patients with severe encephalopathy were not considered in our ventilation/ICU analyses due to near‐perfect correlation between coma and mechanical ventilation requirements.

### 3.2 Multivariable Cox regression analysis and sensitivity analysis in encephalopathy

Encephalopathy was an independent predictor for mortality (HR 2.617, 95% CI 1.481–4.625) in our multivariable Cox regression models based on variables significant in univariable analysis (Table 2). Other significant risk factors for mortality included age, cardiac or cerebrovascular disease and lactate dehydrogenase, while other significant risk factors for ventilation/ICU included hypertension, malignancy, dyspnea, diastolic blood pressure, oxygen saturation, magnesium, lactate dehydrogenase, and neutrophil (Table 2 and Table S5).

**TABLE 2:** Multivariable Cox regression for mortality or ventilation/ICU in clinical characteristics and laboratory parameters of COVID‐19 patients

	*P*	Hazard Ratio	95% CI	
			Lower	Upper
Mortality				
Age	**<0.001**	193.012	40.471	920.495
Hypertension	0.987	0.996	0.610	1.627
Cardiac or cerebrovascular disease	**0.020**	1.566	1.075	2.282
Cough	0.157	0.752	0.507	1.116
Fatigue	0.211	0.791	0.548	1.142
Encephalopathy	**<0.001**	2.617	1.481	4.625
Lactate dehydrogenase	**<0.001**	15.596	5.133	47.388
Ventilation/ICU				
Age	0.957	1.035	0.296	3.623
Hypertension	**0.007**	1.741	1.161	2.612
Diabetes	0.124	1.290	0.933	1.786
Cardiac or cerebrovascular disease	0.407	1.144	0.832	1.573
Chronic kidney disease	0.261	0.806	0.554	1.174
Malignancy	**0.004**	1.718	1.187	2.486
Fever	0.244	1.279	0.846	1.933
Fatigue	0.286	1.177	0.872	1.588
Dyspnea	**<0.001**	2.843	1.808	4.471
Encephalopathy	0.255	1.452	0.764	2.761
Diastolic blood pressure	**<0.001**	0.093	0.025	0.345
Oxygen Saturation	**<0.001**	0.017	0.006	0.052
Blood glucose	0.192	.262	.035	1.964
Blood urea nitrogen	0.172	6.164	0.453	83.859
Creatinine	0.578	2.291	0.123	42.541
Magnesium	**0.031**	6.830	1.190	39.203
Phosphorus	0.188	7.045	0.384	129.302
Lactate dehydrogenase	**<0.001**	8.883	3.373	23.397
White blood cell count	0.308	2.048	0.516	8.125
Neutrophil	**0.015**	4.324	1.325	14.108

Abbreviations: CI, confidence interval; ICU, intensive care unit.

Bold values are statistically significant.

The addition of encephalopathy to a multivariable model comprising other risk factors for adverse outcomes increased the C‐index of the model from 0.78 to 0.81 for mortality and from 0.85 to 0.86 for ventilation/ICU (Table 3).

**TABLE 3:** Measures of model performance in predicting progression to mortality or ventilation/ICU

Variables	Mortality		Ventilation/ICU	
	C‐index	Se	C‐index	Se
Encephalopathy	0.694	0.029	0.606	0.017
Other factors^a^	0.784	0.025	0.851	0.013
Encephalopathy with other factors	0.806	0.022	0.858	0.013

Abbreviations: CI, confidence interval; ICU, intensive care unit.

^a^ Other factors represent age, cardiac or cerebrovascular disease in mortality, and age, diabetes, malignancy, dyspnea in ventilation/ICU.

**FIGURE 2:**
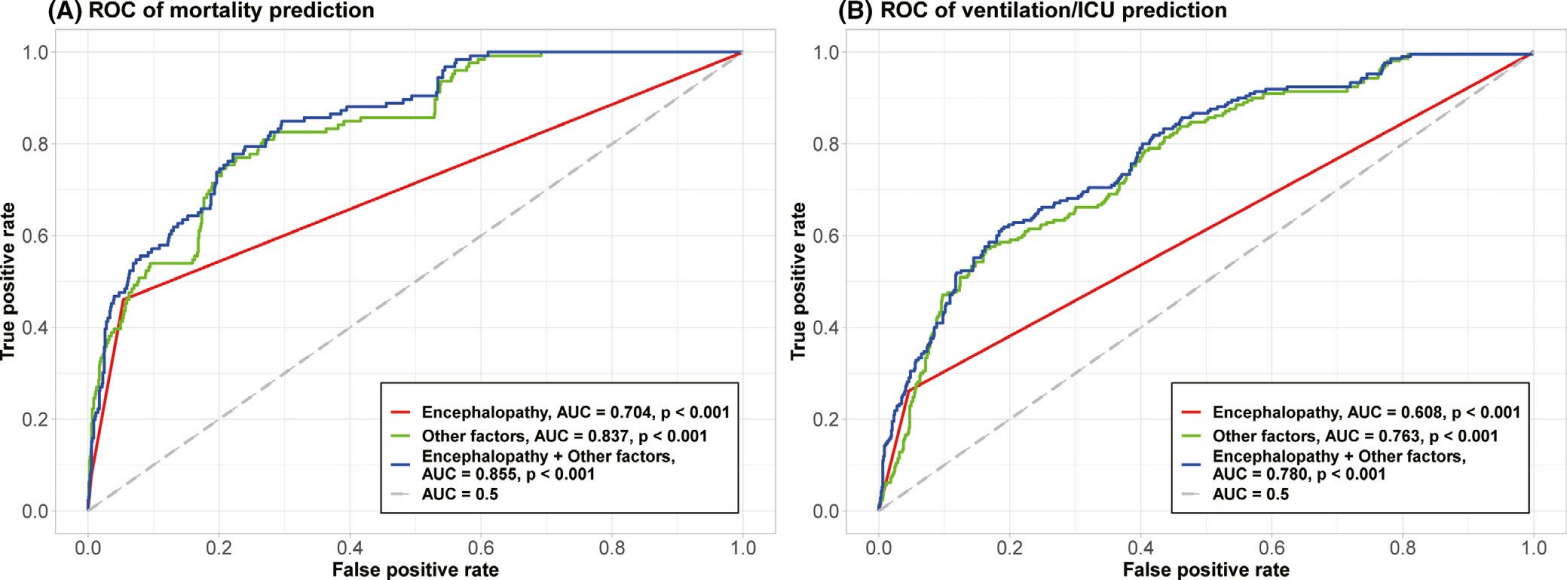
ROC‐AUCs to predict mortality or ventilation/ICU are shown in (A) and (B). In these plots, the x‐axis is false‐positive rate and y‐axis is true‐positive rate. Curves in different colors represent ROC curves based on (1) encephalopathy (2) other factors significant on multivariable Cox regression without encephalopathy (3) encephalopathy +other factors. Other factors represent age, cardiac or cerebrovascular disease, and lactate dehydrogenase in mortality, and hypertension, malignancy, dyspnea, diastolic blood pressure, oxygen saturation, magnesium, lactate dehydrogenase, and neutrophil in ventilation/ICU. ICU, intensive care unit

In addition, the AUCs were higher when encephalopathy was added to other factors to predict mortality (0.86 vs. 0.84, *p *< 0.001, Figure 2a) and ventilation/ICU (0.78 vs. 0.76, *p *< 0.001, Figure 2b).

**FIGURE 3:**
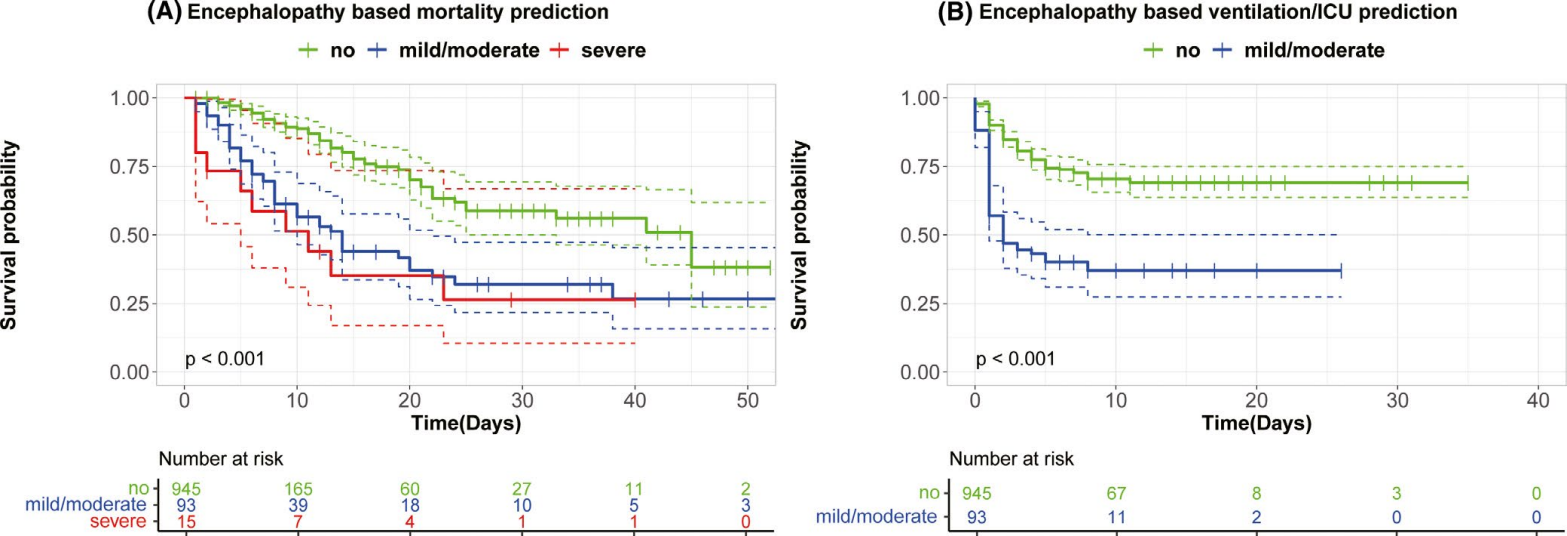
Risk stratification for mortality or ventilation/ICU based on encephalopathy for (A) mortality (B) ventilation/ICU. In these plots, the x‐axis stands for time in days, and the y‐axis is survival probability that represents the probability of not progressing to mortality or ventilation/ICU. ICU, intensive care unit

In a sensitivity analysis, risk stratification survival curves for mortality based on severe encephalopathy (*n* = 15) versus mild/moderate encephalopathy (n=93) vs. no encephalopathy (*n* = 945) at admission were discriminative (*p *< 0.001, Figure 3a). Compared to patients without encephalopathy at admission (*n* = 945), patients with mild/moderate encephalopathy (*n* = 93) had higher rates of mechanical ventilation/ICU admission (*p *< 0.001, Figure 3b).

### 3.3 Radiologic presentations

**FIGURE 4:**
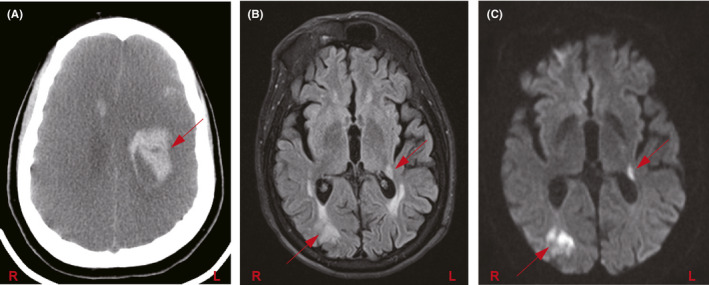
Neurological complications in COVID‐19 patients. (A) Non‐contrast CT head shows diffuse loss of gray‐white differentiation and sulcal effacement, consistent with global hypoxic ischemic encephalopathy. Additionally, there are multifocal intracranial hemorrhages, including a large hematoma in the left fronto‐parietal deep white matter (red arrow); (B) T2‐FLAIR and (C) DWI MRI brain sequences in a patient show right parieto‐occipital and left posterior thalamocapsular acute infarcts (arrows)

Among 174 patients diagnosed with encephalopathy during hospitalization, 68 patients had a brain CT, of which 14 had acute abnormalities. These findings included evidence of acute ischemic stroke in four patients and intracranial hemorrhage in six patients, while four patients had other evidence of focal or global ischemia including decreased attenuation and loss of gray‐white differentiation (Figure 4A). Only four patients had a brain MRI performed in addition to their CT, with MRI confirming ischemic stroke in three patients (Figure 4B and 4C) and revealing an intracranial malignancy in one patient.

## 4 DISCUSSION

It has been widely reported in the literature that varying forms of encephalopathy are common neurological manifestations of COVID‐19, and that patients with severe COVID‐19 tend to present with more neurological symptoms. ^5^, ^10^, ^11^, ^12^, ^15^, ^16^, ^17^ However, these studies do not provide risk assessments based on these symptoms, which would potentially have significant clinical implications. Our study builds upon this prior work by investigating the potential utility of neurologic symptoms in predicting subsequent progression to adverse outcomes, and by building time‐to‐event models that assign risk scores to individual patients.

Our results showed that encephalopathy at admission may be an independent risk factor for later progression to death in COVID‐19 patients and that it improved the predictive accuracy of our risk factor‐based models with its addition. We also found that coma, as a marker of severe encephalopathy, provided further risk stratification and was associated with a significantly higher rate of mortality in our survival analyses. Our findings suggest that healthcare providers may consider encephalopathy and coma as key factors for the purposes of risk stratification, potentially signaling COVID‐19 patients at high risk of developing adverse outcomes. In combination with other factors, this may help inform their decisions about immediate urgency of care and longer‐term resource utilization. However, as with risk stratification in other diseases, caution must be taken that this information does not lead to a self‐fulfilling prophecy.

Other independent indicators included laboratory parameters like lactate dehydrogenase for death, and diastolic blood pressure, oxygen saturation, magnesium, lactate dehydrogenase, neutrophil for ventilation/ICU. That is understandable because patients with SARS‐CoV‐2 infection often have major organ dysfunction. Similarly, our finding that encephalopathy at admission is an independent risk factor for later progression to death may support the proposition that the CNS is directly involved in SARS‐CoV‐2 infection.

Accumulating evidence indicates that neurological complications of SARS‐CoV‐2 are highly related to immune system malfunction. ^18^, ^19^, ^20^, ^21^, ^22^ Overreacting of the innate immune system results in the uncontrolled release of cytokines and chemokines in patients with severe COVID‐19, leading to vascular system dysfunction and consequent brain‐blood barrier (BBB) disruption, providing a path for inflammatory mediators, immune cells, and virus particles to access the CNS. ^23^, ^24^, ^25^, ^26^, ^27^ It is proposed that the overproduction of inflammatory cytokines in SARS‐CoV‐2 infection may lead to inflammatory damage in the brain tissue, explaining the appearance of non‐specific complications, including headache, dizziness, taste, and smell dysfunctions. ^21^, ^27^, ^28^ However, as we observed, it is possible that the neurologic manifestations associated with COVID‐19 may be directly related to the infection, with encephalitis induced by hematogenous spread or neuronal retrograde transport as potential mechanisms. ^15^, ^29^, ^30^ These neurological manifestations have been demonstrated in other CoV infections such as SARS‐CoV and MERS‐CoV, which have provided strong evidence for CoV neuroinvasive capacity. ^21^, ^31^, ^32^, ^33^ Several reports have documented CSF samples that were positive for SARS‐CoV RNA. Also, there is evidence of monocyte and lymphocyte infiltrations in the brain, ischemic changes of neurons, and demyelinating abnormalities. ^34^, ^35^ Thus, it is proposed that neurological complications of COVID‐19 may include a rare direct infection of nerve ends. Several studies have described COVs, particularly SARS‐CoV‐2, as neurotropic viruses with neuroinvasive capabilities that directly invade the CNS through neuronal retrograde routes and result in neurological pathologies such as encephalomyelitis. ^12^, ^36^, ^37^, ^38^ Detection of SARS‐CoV‐2 and other CoVs, particularly SARS‐CoV in the cerebrospinal fluid (CSF) of infected patients, provides additional support to the potential neuroinvasive contribution of SARS‐CoV‐2. ^6^, ^37^, ^39^, ^40^, ^41^, ^42^, ^43^ Moreover, the endothelial cells of BBB may act as a bed for SARS‐CoV‐2 accession to CNS through angiotensin‐converting enzyme 2 (ACE2).

This study has several limitations. First, this was a retrospective single‐center study. Second, most of the symptoms used in our analysis were patients’ subjective descriptions as recorded via chart review, and most initial examinations were not performed by a neurologist or other clinician with neurologic expertise. It is likely that patients with encephalopathy were often too confused or stuporous to be able to report additional symptoms, which may have been underreported. Third, as many patients might be delayed for admission in the pandemic, the timing when they developed neurological symptoms could not be revealed by this study. Besides, we had limited data on advanced neurologic testing such as magnetic resonance imaging, lumbar puncture, and electromyography/nerve conduction velocity, because these tests were often purposefully avoided in COVID‐19 patients to reduce the risk of cross infection. Future prospective multi‐center studies are needed to confirm and expand on our findings, with additional testing to explore the neurologic implications of COVID‐19.

## 5 CONCLUSION

Encephalopathy at admission predicts later progression to death in COVID‐19 patients, which may have important implications for risk stratification in clinical practice.

## IRB

Institutional Review Boards of Hospital of University of Pennsylvania was obtained for the study cohort with waiver of consent, with confirmation number (dbhbeegg) and protocol number (843448).

## CONFLICT OF INTEREST

The authors declare that they have no conflict of interest.

## Supporting information

Supplementary Material

## Data Availability

Data are not made publicly available due to HIPAA regulations, but anonymized data can be sent to the corresponding author by reasonable requests.
