# Supplementary material for: Encephalopathy at admission predicts adverse outcomes in patients with SARS‐CoV‐2 infection
Source: CNS Neurosci Ther. 2021 Jun 16;27(10):1127–35. doi: 10.1111/cns.13687 (PMC8444722; doi:10.1111/cns.13687)
Supplement: Supplementary file 1 — Supplementary Material [file CNS-27-1127-s001.docx]

eTable 1. Type of admission notes mentioning encephalopathy in COVID-19 patients

| **Encephalopathy** | **At admission**  **(n= 108)** | |
| --- | --- | --- |
|  | **Coma**  **(n= 15)** | **No coma**  **(n= 93)** |
| Notes |  |  |
| Emergency medicine | 14 | 89 |
| ED triage note | 0 | 1 |
| Trauma service | 1 | 0 |
| Cardiac surgery | 0 | 1 |
| Cardiovascular medicine | 0 | 1 |
| Medicine | 0 | 1 |

Abbreviations: ED, Emergency Department

eTable 2. Clinical characteristics of 1053 COVID-19 patients with and without encephalopathy during the entire hospitalization

| Clinical and laboratory | Total (n= 1053) | Encephalopathy  (n= 174) | No encephalopathy  (n= 879) | P value |
| --- | --- | --- | --- | --- |
| Clinical characteristic, No. % |  |  |  |  |
| Age, mean (SD), y | 52.4 (20.2) | 70.2 (15.9) | 48.8 (19.0) |  |
| <50 | 487 (46.2%) | 17 (9.8%) | 470 (53.5%) | **<.001** |
| ≥50 | 566 (53.8%) | 157 (90.2%) | 409 (46.5%) |  |
| Sex |  |  |  |  |
| Male | 505 (48.0) | 86 (49.4%) | 419 (47.7%) | .672 |
| Female | 548 (52.0) | 88 (50.6%) | 460 (52.3%) |  |
| Comorbidities |  |  |  |  |
| Hypertension | 567 (53.8) | 136 (78.2%) | 431 (49.0%) | **<.001** |
| Diabetes | 349 (33.1) | 81 (46.6%) | 268 (30.5%) | **<.001** |
| Cardiac or cerebrovascular disease | 249 (23.6) | 89 (51.1%) | 160 (18.2%) | **<.001** |
| Chronic kidney disease | 164 (15.6) | 53 (30.5%) | 111 (12.6%) | **<.001** |
| Malignancy | 107 (10.2) | 31 (17.8%) | 76 (8.6%) | **<.001** |
| Typical symptoms |  |  |  |  |
| Cough | 788 (74.8) | 115 (66.1%) | 673 (76.5%) | **.004** |
| Fever | 776 (73.7) | 148 (85.1%) | 628 (71.4%) | **<.001** |
| Dyspnea | 712 (67.6) | 135 (77.6%) | 577 (65.6%) | **.002** |
| Fatigue | 448 (42.5) | 73 (42.0%) | 375 (42.7%) | .863 |
| Chill | 365 (34.7) | 38 (21.8%) | 327 (37.2%) | **<.001** |
| Nausea | 283 (26.9) | 36 (20.7%) | 247 (28.1%) | **.044** |
| Diarrhoea | 271 (25.7) | 33 (19.0%) | 238 (27.1%) | **.025** |
| Chest pain | 250 (23.7) | 31 (17.8%) | 219 (24.9%) | **.044** |
| Throat pain | 246 (23.4) | 20 (11.5%) | 186 (25.7%) | **.003** |
| Anorexia | 218 (20.7) | 30 (17.2%) | 188 (21.4%) | .217 |
| Abdominal pain | 170 (16.1) | 19 (10.9%) | 152 (17.3%) | **.037** |
| Nervous system symptoms |  |  |  |  |
| Headache | 195 (18.5) | 22 (12.3%) | 173 (19.7%) | **.029** |
| Dizziness | 88 (8.4) | 22 (12.6%) | 66 (7.5%) | **.025** |
| Seizure | 33 (3.1) | 18 (10.3%) | 15 (1.7%) | **<.001** |
| Acute cerebrovascular disease | 15 (1.4) | 12 (6.9%) | 3 (0.3%) | **<.001** |
| Ataxia | 8 (0.8) | 6 (1.5%) | 2 (0.5%) | **<.001** |
| Taste impairment | 42 (4.0) | 1 (0.6%) | 41 (4.7%) | **.012** |
| Smell impairment | 42 (4.0) | 1 (0.6%) | 41 (4.7%) | **.012** |
| Vision impairment | 6 (0.6) | 2 (1.1%) | 4 (0.5%) | .575 |
| Outcomes |  |  |  |  |
| Mortality | 126 (12.0) | 77 (44.3%) | 49 (5.6%) | **<.001** |
| Ventilation/ICU | 221 (21.0) | 110 (63.8) | 111 (12.6%) | **<.001** |

Abbreviations: SD, Standard deviation. ICU, intensive care unit.

eTable 3. Laboratory parameters of 495 COVID-19 patients with and without encephalopathy during the entire hospitalization

| Laboratory parameters,  median (range) | Total (n= 495) | Encephalopathy  (n= 43) | No encephalopathy  (n= 452) | P value |
| --- | --- | --- | --- | --- |
| Blood pressure, mmHg |  |  |  |  |
| Systolic blood pressure | 124.0 (73.0-247.0) | 128.0 (87.0-197.0) | 123.0 (73.0-247.0) | .057 |
| Diastole blood pressure | 79.0 (36.0-136.0) | 76.0 (43.0-130.0) | 79.0 (36.0-136.0) | .232 |
| Blood oxygen saturation, % | 96.0 (59.0-100.0) | 97.0 (81.0-100.0) | 96.0 (59.0-100.0) | .098 |
| Blood glucose, mg/dL | 106.0 (9.0-1258.0) | 129.0 (80.0-1258.0) | 102.5 (9.0-461.0) | **<.001** |
| Blood urea nitrogen, mg/dL | 15.0 (2.0-206.0) | 22.0 (7.0-206.0) | 14.0 (2.0-191.0) | **<.001** |
| Creatinine, mg/dL | 1.0 (0.3-28.4) | 1.2 (0.5-27.2) | 1.0 (0.3-28.4) | **.001** |
| Sodium, mmol/L | 136.0 (118.0-169.0) | 136.0 (118.0-169.0) | 136.0 (125.1-150.0) | .208 |
| Potassium, mmol/L | 4.0 (2.5-10.0) | 4.3 (2.6-10.0) | 3.9 (2.5-6.1) | **.001** |
| Chloride, mmol/L | 100.0 (9.4-126.0) | 97.0 (9.4-126.0) | 100.0 (9.6-122.7) | .129 |
| Calcium, mmol/L | 8.2 (2.0-10.2) | 8.5 (7.2-9.8) | 7.9 (2.0-10.2) | **<.001** |
| Magnesium, mmol/L | 1.7 (0.8-4.7) | 2.0 (1.2-4.7) | 1.6 (0.8-2.7) | **<.001** |
| Phosphorus, mmol/L | 2.3 (0.8-14.9) | 3.5 (2.3-10.9) | 1.5 (0.8-14.9) | **<.001** |
| Protein Total, g/dL | 6.8 (4.6-8.7) | 7.2 (6.2-8.5) | 6.8 (4.6-8.7) | **.036** |
| Albumin, g/dL | 3.9 (2.4-5.1) | 3.6 (2.4-4.8) | 3.9 (2.7-5.1) | **.011** |
| Bilirubin Total, mg/dL | 0.9 (0.2-3.0) | 0.4 (0.2-2.0) | 1.0 (0.2-3.0) | **<.001** |
| Bilirubin Direct, mg/dL | 0.2 (0.1-1.1) | 0.1 (0.1-1.1) | 0.2 (0.1-0.5) | **.003** |
| Alkaline Phosphatase, U/L | 93.0 (25.0-458.0) | 70.0 (32.0-240.0) | 94.0 (25.0-458.0) | **.011** |
| AST, U/L | 31.0 (7.0-420.0) | 37.0 (8.0-208.0) | 31.0 (7.0-420.0) | .148 |
| ALT, U/L | 23.5 (6.0-416.0) | 21.5 (6.0-91.0) | 24.0 (6.0-416.0) | .389 |
| Creatine kinase, U/L | 158.5 (0.4-4972.0) | 345.0 (84.0-1167.0) | 154.0 (0.4-4972.0) | **.005** |
| C-reactive protein, mg/dL | 8.8 (0.1-163.1) | 13.8 (0.8-53.0) | 7.9 (0.1-163.1) | **.018** |
| Lactate dehydrogenase, U/L | 262.5 (105.0-1021.0) | 286.5 (140.0-864.0) | 256.5 (105.0-1021.0) | .129 |
| NT-proBNP, pg/mL | 327.5 (5.0-44800.0) | 1028.0 (82.0-44800.0) | 171.5 (5.0-3463.0) | **.007** |
| White blood cell, ×10^9^/L | 6.6 (1.1-27.6) | 9.0 (2.8-27.6) | 6.4 (1.1-18.2) | **<.001** |
| Hemoglobin, g/dL | 13.5 (5.7-18.8) | 12.0 (7.1-18.8) | 13.5 (5.7-18.8) | **.004** |
| Platelet, ×10^9^/L | 207.0 (0.6-514.0) | 209.5 (18.9-514.0) | 204.0 (0.6-512.0) | .575 |
| Neutrophil, ×10^9^/L | 4.8 (0.7-177.0) | 6.7 (1.9-24.4) | 4.7 (0.7-177.0) | **<.001** |
| Lymphocyte, ×10^9^/L | 1.2 (0.1-9.6) | 0.9 (0.2-3.9) | 1.2 (0.1-9.6) | **.031** |
| D-dimer, mg/L | 0.8 (0.1-64.9) | 1.3 (0.4-64.5) | 0.7 (0.1-64.9) | **.019** |

Abbreviations: AST, Aspartate Transaminase. ALT, Alanine aminotransferase.

eTable 4. Univariable Cox regression for mortality or ventilation/ICU in clinical characteristics of COVID-19 patients

| Clinical characteristic, No. % | Total  (n= 1053) | Mortality  (n= 126) | No mortality  (n= 927) | Hazard Ratio  (95% CI) | P value | Vent/ICU  (n= 221) | No Vent/ICU  (n= 832) | Hazard Ratio  (95% CI) | P value |
| --- | --- | --- | --- | --- | --- | --- | --- | --- | --- |
| Age |  |  |  |  |  |  |  |  |  |
|  |  |  |  |  |  |  |  |  |  |
| <50 | 487 (46.2) | 6 (4.8) | 481 (51.9) |  | **.001** | 32 (14.5) | 455 (54.1) |  |  |
| ≥50 | 566 (53.8) | 120 (95.2) | 446 (48.1) | 4.213 (1.841-9.642) |  | 189 (85.5) | 377 (45.9) | 3.958 (2.709-5.782) | **<.001** |
| Sex |  |  |  |  |  |  |  |  |  |
| Male | 505 (48.0) | 59 (46.8) | 446 (48.1) | .714 (.502-1.017) | .062 | 117 (52.9) | 388 (46.6) | 1.236 (.949-1.609) | .117 |
| Female | 548 (52.0) | 67 (53.2) | 481 (51.9) |  |  | 104 (47.1) | 444 (53.4) |  |  |
| Comorbidities |  |  |  |  |  |  |  |  |  |
| Hypertension | 567 (53.8) | 104 (82.5) | 463 (49.9) | 1.713 (1.078-2.721) | **.023** | 175 (79.2) | 392 (47.1) | 2.789 (2.012-3.866) | **<.001** |
| Diabetes | 349 (33.1) | 68 (54.0) | 281 (30.3) | 1.191 (.837-1.694) | .331 | 120 (54.3) | 229 (27.5) | 2.100 (1.610-2.741) | **<.001** |
| Cardiac or cerebrovascular disease | 249 (23.6) | 71 (56.3) | 178 (19.2) | 2.120 (1.488-3.020) | **<.001** | 93 (42.1) | 156 (18.8) | 2.004 (1.531-2.622) | **<.001** |
| Chronic kidney disease | 164 (15.6) | 36 (28.6) | 128 (13.8) | 1.109 (.753-1.635) | .600 | 57 (25.8) | 107 (12.9) | 1.574 (1.162-2.132) | **.003** |
| Malignancy | 107 (10.2) | 27 (21.4) | 80 (8.6) | 1.212 (.790-1.859) | .379 | 42 (19.0) | 122 (16.8) | 1.793 (1.279-2.514) | **.001** |
| Typical symptoms |  |  |  |  |  |  |  |  |  |
| Cough | 788 (74.8) | 87 (69.0) | 701 (75.6) | .626 (.428-.915) | **.016** | 170 (76.9) | 618 (73.0) | 1.090 (.797-1.490) | .591 |
| Fever | 776 (73.7) | 110 (87.3) | 666 (71.8) | 1.105 (.650-1.879) | .711 | 188 (85.1) | 588 (70.7) | 1.769 (1.221-2.564) | **.003** |
| Dyspnea | 712 (67.6) | 107 (84.9) | 605 (65.3) | 1.272 (.77-2.081) | .339 | 196 (88.7) | 516 (62.0) | 3.619 (2.386-5.488) | **<.001** |
| Fatigue | 448 (42.5) | 52 (41.3) | 396 (42.7) | .656 (.460-.937) | **.021** | 123 (55.7) | 325 (39.1) | 1.614 (1.237-2.105) | **<.001** |
| Chill | 365 (34.7) | 34 (27.0%) | 331 (35.7) | .892 (.602-1.323) | .571 | 67 (30.3) | 298 (35.8) | .864 (.648-1.151) | .371 |
| Nausea | 283 (26.9) | 27 (21.4) | 256 (27.6) | .690 (.451-1.057) | .088 | 66 (29.9) | 217 (26.1) | 1.153 (.864-1.538) | .334 |
| Diarrhoea | 271 (25.7) | 31 (24.6) | 240 (25.9) | .681 (.454-1.023) | .064 | 69 (31.2) | 202 (24.3) | 1.209 (.910-1.608) | .191 |
| Chest pain | 250 (23.7) | 29 (23.0) | 221 (23.8) | .958 (.632-1.451) | .838 | 62 (28.1) | 188 (22.6) | 1.319 (.983-1.769) | .065 |
| Throat pain | 246 (23.4) | 22 (17.5) | 224 (24.2) | .800 (.505-1.268) | .342 | 46 (20.8) | 200 (24.0) | .886 (.640-1.226) | .465 |
| Anorexia | 218 (20.7) | 22 (17.5) | 196 (21.1) | .728 (.459-1.153) | .176 | 57 (25.8) | 161 (19.4) | 1.312 (.970-1.773) | .078 |
| Abdominal pain | 170 (16.1) | 17 (13.5) | 154 (16.6) | .818 (.491-1.365) | .443 | 36 (16.3) | 135 (16.2) | 1.002 (.701-1.432) | .991 |
| Nervous system symptoms |  |  |  |  |  |  |  |  |  |
| Headache | 172 (16.3) | 11 (8.7) | 161 (17.4) | .642 (.346-1.192) | .160 | 34 (15.4) | 138 (16.6) | 1.077 (.747-1.555) | .690 |
| Encephalopathy | 108 (10.3) | 58 (46.0) | 50 (5.4) |  |  | 66 (29.9) | 42 (5.0) |  |  |
| Mild/moderate | 93 (8.8) | 48 (38.1) | 45 (4.9) | 3.005 (2.071-4.360) | **<.001** | 55 (24.9) | 38 (4.6) | 3.333 (2.447-4.539) | **<.001** |
| Severe | 15 (1.4) | 5 (4.0) | 10 (1.1) | 3.895 (1.997-7.594) | **<.001** | NA | NA | NA | NA |
| Dizziness | 66 (6.3) | 7 (5.6) | 59 (6.4) | .716 (.334-1.536) | .391 | 17 (7.7) | 49 (5.9) | 1.129 (.688-1.852) | .631 |
| Seizure | 24 (2.3) | 3 (2.4) | 21 (2.3) | .448 (.142-1.409) | .170 | 7 (3.2) | 17 (2.0) | .976 (.459-2.078) | .950 |
| Acute cerebrovascular disease | 4 (0.4) | 0 (NA) | 4 (0.4) | .049 (.000-758.673) | .540 | 2 (0.9) | 2 (0.2) | 2.194 (.545-8.838) | .269 |
| Ataxia | 6 (0.6) | 2 (1.6) | 4 (0.4) | 1.516 (.374-6.139) | .560 | 1 (0.5) | 5 (0.6) | .535 (.075-3.817) | .532 |
| Taste impairment | 40 (3.8) | 1 (0.8) | 39 (4.2) | .350 (.049-2.506) | .296 | 3 (1.4) | 37 (4.4) | .387 (.124-1.208) | .102 |
| Smell impairment | 41 (3.9) | 1 (0.8) | 40 (4.3) | .394 (.055-2.825) | .354 | 2 (0.9) | 39 (4.7) | .264 (.065-1.062) | .061 |
| Vision impairment | 2 (0.2) | 0 (NA) | 2 (0.2) | .050 (.000-NA) | .943 | 0 (NA) | 2 (0.2) | .050 (.000-NA) | .721 |

Abbreviations: Vent, ventilation. ICU, intensive care unit.

eTable 5. Univariate Cox regression for mortality or ventilation/ICU in laboratory parameters of COVID-19 patients

| Laboratory parameters, median (range) | Total  (n= 495) | Mortality  (n= 55) | No mortality  (n= 440) | Hazard Ratio  (95% CI) | P value | Vent/ICU  (n= 120) | No Vent/ICU  (n= 375) | Hazard Ratio  (95% CI) | P value |
| --- | --- | --- | --- | --- | --- | --- | --- | --- | --- |
| Blood pressure, mmHg |  |  |  |  |  |  |  |  |  |
| Systolic blood pressure | 124.0 (73.0-247.0) | 114.0 (79.0-197.0) | 125.0 (73.0-247.0) | .991 (.978 -1.004) | .158 | 123.0 (73.0-247.0) | 124.0 (81.0-200.0) | .997 (.988 -1.005) | .472 |
| Diastolic blood pressure | 79.0 (36.0-136.0) | 75.0 (36.0-115.0) | 79.0 (42.0-136.0) | .999 (.982 -1.016) | .894 | 75.0 (36.0-136.0) | 79.0 (49.0-135.0) | .973 (.959 -.986) | **<.001** |
| Blood oxygen saturation, % | 96.0 (59.0-100.0) | 93.0 (59.0-100.0) | 96.0 (68.0-100.0) | .996 (.961 -1.032) | .812 | 94.0 (59.0-100.0) | 97.0 (88.0-100.0) | .927 (.906 -.949) | **<.001** |
| Blood glucose, mg/dL | 106.0 (9.0-1258.0) | 116.0 (71.0-247.0) | 105.0 (9.0-1258.0) | .997 (.991-1.003) | .304 | 118.0 (50.0-1258.0) | 102.0 (9.0-404.0) | 1.002 (1.001-1.003) | **<.001** |
| Blood urea nitrogen, mg/dL | 15.0 (2.0-206.0) | 20.0 (7.0-89.0) | 14.0 (2.0-206.0) | .993 (.977 -1.009) | .389 | 18.0 (2.0-206.0) | 14.0 (4.0-88.0) | 1.010 (1.004-1.016) | **.001** |
| Creatinine, mg/dL | 1.0 (0.3-28.4) | 1.2 (0.4-9.8) | 1.0 (0.3-28.4) | .917 (.750 -1.120) | .395 | 1.1 (0.4-28.4) | 1.0 (0.3-10.3) | 1.063 (1.013-1.115) | **.013** |
| Sodium, mmol/L | 136.0 (118.0-169.0) | 135.0 (125.1-164.0) | 136.0 (118.0-169.0) | .990 (.945-1.037) | .678 | 136.0 (118.0-169.0) | 136.0 (125.4-164.0) | 1.003 (.970-1.038) | .841 |
| Potassium, mmol/L | 4.0 (2.5-10.0) | 4.2 (2.9-5.4) | 4.0 (2.5-10.0) | .910 (.612 -1.353) | .641 | 4.0 (2.6-10.0) | 3.9 (2.5-5.6) | 1.296 (1.045-1.608) | **.018** |
| Chloride, mmol/L | 100.0 (9.4-126.0) | 99.0 (83.5-124.0) | 100.0 (9.4-126.0) | 1.022 (.991-1.053) | .174 | 99.0 (9.4-126.0) | 100.3 (9.6-124.0) | .993 (.976-1.009) | .384 |
| Calcium, mmol/L | 8.2 (2.0-10.2) | 2.9 (2.1-9.4) | 8.3 (2.0-10.2) | .952 (.864-1.050) | .326 | 8.0 (2.0-9.7) | 8.3 (2.0-10.2) | 1.027 (.965-1.093) | .402 |
| Magnesium, mmol/L | 1.7 (0.8-4.7) | 1.2 (0.8-2.8) | 1.8 (0.8-4.7) | .533 (.246 -1.154) | .110 | 1.9 (0.8-4.7) | 1.6 (0.8-2.4) | 2.321 (1.517-3.553) | **<.001** |
| Phosphorus, mmol/L | 2.3 (0.8-14.9) | 1.4 (0.8-6.3) | 2.4 (0.9-14.9) | .852 (.646 -1.122) | .254 | 3.1 (0.8-14.9) | 1.5 (0.9-5.4) | 1.167 (1.085-1.256) | **<.001** |
| Protein Total, g/dL | 6.8 (4.6-8.7) | 6.7 (4.6-8.4) | 6.9 (4.7-8.7) | .930 (.670-1.290) | .663 | 6.7 (4.6-8.7) | 6.9 (4.7-8.6) | .919 (.742 -1.139) | .440 |
| Albumin, g/dL | 3.9 (2.4-5.1) | 3.6 (2.4-5.1) | 3.9 (2.7-5.1) | .609 (.363-1.022) | .060 | 3.7 (2.4-5.1) | 3.9 (2.7-5.1) | .857 (.615 -1.194) | .362 |
| Bilirubin Total, mg/dL | 0.9 (0.2-3.0) | 1.3 (0.2-2.2) | 0.8 (0.2-3.0) | 1.498 (.923-2.430) | .102 | 0.8 (0.2-2.2) | 0.9 (0.2-3.0) | .821 (.592-1.138) | .236 |
| Bilirubin Direct, mg/dL | 0.2 (0.1-1.1) | 0.3 (0.1-1.0) | 0.2 (0.1-1.1) | 3.410 (.640-18.163) | .151 | 0.2 (0.1-1.1) | 0.2 (0.1-0.5) | 1.038 (.251-4.304) | .959 |
| Alkaline Phosphatase, U/L | 93.0 (25.0-458.0) | 117.5 (32.0-262.0) | 91.5 (25.0-458.0) | 1.001 (.996-1.005) | .791 | 94.0 (31.0-429.0) | 93.0 (25.0-458.0) | 1.001 (.998-1.005) | .407 |
| AST, U/L | 31.0 (7.0-420.0) | 36.0 (12.0-208.0) | 31.0 (7.0-420.0) | .998 (.989-1.007) | .660 | 40.5 (8.0-208.0) | 29.0 (7.0-420.0) | 1.001 (.997-1.006) | .561 |
| ALT, U/L | 23.5 (6.0-416.0) | 23.0 (7.0-91.0) | 24.0 (6.0-416.0) | .995 (.984 -1.007) | .414 | 26.0 (6.0-106.0) | 23.0 (6.0-416.0) | .999 (.994-1.005) | .778 |
| Creatine kinase, U/L | 158.5 (0.4-4972.0) | 171.5 (11.0-969.0) | 158.0 (0.4-4972.0) | .999 (.998 -1.001) | .428 | 178.0 (11.0-1167.0) | 153.0 (0.4-4972.0) | 1.000 (.999-1.000) | .672 |
| C-reactive protein, mg/dL | 8.8 (0.1-163.1) | 14.2 (0.3-53.0) | 7.0 (0.1-163.1) | .998 (.981 -1.015) | .804 | 13.3 (0.2-163.1) | 5.1 (0.1-160.0) | 1.004 (.996-1.011) | .338 |
| Lactate dehydrogenase, U/L | 262.5 (105.0-1021.0) | 345.0 (109.0-987.0) | 254.0 (105.0-1021.0) | 1.002 (1.000-1.003) | **.034** | 308.0 (109.0-910.0) | 237.0 (105.0-1021.0) | 1.002 (1.000-1.003) | **.004** |
| NT-proBNP, pg/mL | 327.5 (5.0-44800.0) | 900.0 (82.0-44800.0) | 275.0 (5.0-35000.0) | 1.000 (1.000-1.000) | .202 | 431.0 (23.0-35000.0) | 171.0 (5.0-44800.0) | 1.000 (1.000-1.000) | .446 |
| White blood cell, ×10^9^/L | 6.6 (1.1-27.6) | 6.5 (1.1-23.2) | 6.6 (1.8-27.6) | .953 (.882-1.029) | .214 | 7.6 (1.1-23.2) | 6.2 (2.2-27.6) | 1.088 (1.046-1.131) | **<.001** |
| Hemoglobin, g/dL | 13.5 (5.7-18.8) | 12.0 (6.8-18.8) | 13.5 (5.7-18.8) | .929 (.822 -1.050) | .240 | 13.1 (6.8-18.8) | 13.6 (5.7-18.8) | .955 (.885 -1.031) | .241 |
| Platelet, ×10^9^/L | 207.0 (0.6-514.0) | 194.0 (0.6-450.0) | 208.0 (1.2-514.0) | .998 (.995 -1.001) | .207 | 201.0 (0.6-514.0) | 207.0 (4.0-512.0) | 1.000 (.998-1.002) | .913 |
| Neutrophil, ×10^9^/L | 4.8 (0.7-177.0) | 6.2 (0.7-146.0) | 4.6 (0.7-177.0) | 1.002 (.989-1.015) | .743 | 6.4 (0.7-177.0) | 4.4 (0.7-24.4) | 1.010 (1.003-1.017) | **.006** |
| Lymphocyte, ×10^9^/L | 1.2 (0.1-9.6) | 1.0 (0.1-8.9) | 1.2 (0.2-9.6) | 1.085 (.953-1.237) | .219 | 1.0 (0.1-9.6) | 1.2 (0.3-9.3) | 1.020 (.915-1.138) | .717 |
| D-dimer, mg/L | 0.8 (0.1-64.9) | 0.8 (0.2-14.3) | 0.8 (0.1-64.9) | .974 (.924-1.027) | .334 | 0.8 (0.1-64.9) | 0.7 (0.1-21.8) | 1.000 (.986-1.013) | .979 |

Abbreviations: SD, Standard deviation. ICU, intensive care unit. AST, Aspartate Transaminase. ALT, Alanine aminotransferase.
